# Dual-Task Training Program for Older Adults: Blending Gait, Visuomotor and Cognitive Training

**DOI:** 10.3389/fnetp.2021.736232

**Published:** 2021-09-29

**Authors:** Akshata Nayak, Rehab Alhasani, Anuprita Kanitkar, Tony Szturm

**Affiliations:** ^1^ Department of Physical Therapy, College of Rehabilitation Sciences, University of Manitoba, Winnipeg, MB, Canada; ^2^ School of Physical and Occupational Therapy, Faculty of Medicine, McGill University, Montreal, QC, Canada; ^3^ Department of Rehabilitation Sciences, College of Health and Rehabilitation Sciences, Princess Nourah Bint Abdulrahman University, Riyadh, Saudi Arabia

**Keywords:** aging, game-assisted dual-task training, treadmill, recumbent bicycle, spatial-temporal gait variables, executive cognitive function

## Abstract

**Objective:** Physical and cognitive impairments are common with aging and often coexist. Changes in the level of physical and mental activity are prognostic for adverse health events and falls. Dual-task (DT) training programs that can improve mobility and cognition simultaneously can bring significant improvements in rehabilitation. The objective of this mixed methods exploratory RCT was to provide evidence for the feasibility and therapeutic value of a novel game-assisted DT exercise program in older adults.

**Methods:** Twenty-two community dwelling participants, between the ages of 70–85 were randomized to either dual-task treadmill walking (DT-TR) or dual-task recumbent bicycle (DT-RC). Both groups viewed a standard LED computer monitor and performed a range of cognitive game tasks while walking or cycling; made possible with the use of a “hands-free”, miniature, inertial-based computer mouse. Participants performed their respective 1-h DT exercise program twice a week, for 12 weeks at a community fitness centre. Semi-structured interviews and qualitative analysis was conducted to evaluate the participant’s experiences with the exercise program. Quantitative analysis included measures of standing balance, gait function (spatiotemporal gait variable), visuomotor and executive cognitive function, tested under single and DT walking conditions.

**Results:** Compliance was 100% for all 22 participants. Four themes captured the range of participant’s experiences and opinions: 1) reasons for participation, 2) difficulties with using the technologies, 3) engagement with the computer games, and 4) positive effects of the program. Both groups showed significant improvements in standing balance performance, visuomotor and visuospatial executive function. However, significant improvement in dual task gait function was observed only in the DT-TR group. Medium to large effect sizes were observed for most balance, spatiotemporal gait variables, and cognitive performance measure.

**Conclusion:** With only minor difficulties with the technology being reported, the findings demonstrate feasible trial procedures and acceptable DT oriented training with a high compliance rate and positive outcomes. These findings support further research and development, and will direct the next phase of a full-scale RCT.

**Registration information:** This clinical trial has been registered at ClinicalTrials.gov Protocol Registration System: NCT01940055.

## Introduction

For older adults, community ambulation is strongly associated with the preservation of skills for independent living, social participation, and healthy aging (Hirvensalo et al., 2000; Onder et al., 2005; Simonsick et al., 2005). Independent community walking requires both mobility skills and cognitive flexibility, to safely manage environmental demands. These include, but not limited to, navigating crowded environments, managing complex terrains, attending to and tracking visual targets, reading, etc. Mobility and cognitive abilities are closely linked (Muir et al., 2012; Allali et al., 2016), and it is now well established that dual-task walking results in significant gait changes among older adults (Montero-Odasso et al., 2012; Nankar et al., 2017). There is a decline in both mobility skills and cognition with increase in age. The rate of this decline is prognostic of future adverse health events such as falling (Yamada et al., 2011; Mirelman et al., 2012).

Studies have shown that increased physical activity improves mobility and reduces the risk of injuries among older adults (Lord et al., 2003; VanSwearingen et al., 2011; Freiberger et al., 2012). There are also reports of improved Executive Cognitive Function (ECF) with physical activity (Verdelho et al., 2012; Gates et al., 2013). Studies that are more recent have used multimodal interventions, combining motor and cognitive training, to improve both mobility skills and ECF (Gregory et al., 2017; Conradsson and Halvarsson, 2019). In this field of multimodal interventions, the application of digital media and computer technology provides a number of promising approaches. Anderson-Hanley et al. (2012) showed that coupling recumbent cycling exercise with a virtual reality task enhanced ECF and level of cognitive impairment more than aerobic exercise alone. Another promising approach involves virtual reality simulations viewed during treadmill walking (Feasel et al., 2011; Shema et al., 2014; Mirelman et al., 2016). Results showed reduced incidence of falls among older adults. This provides a task-specific approach to dual-task walking training.

Maximizing participation is also a major goal of interventions. An emerging approach is to combine exercise and activities with computer games, making training a more engaging experience (Szturm et al., 2011; Zeng et al., 2017; Montero-Alía et al., 2019). Additionally, digital media in the form of computer games can also challenge and train many different aspects of ECF, expanding their scope of use (Rebok et al., 2014; Strenziok et al., 2014).

Based on this information, a Game-based Rehabilitation Platform (GRP) has been developed (Szturm et al., 2013; Szturm et al., 2017) that consists of a treadmill, display monitor, and an interactive computer game subsystem. The GRP provides an integrated approach in treating the decline in balance, gait, visuomotor skills, and ECF, that is associated with aging. The purpose of this study is to provide evidence for the feasibility of conducting a full-scale RCT using the GRP for DT gait training in older adults. Feasibility was used to evaluate the implementation, safety, acceptance, and retention/compliance of this new training program in a community centre. Some of this was done by usual observation and some by the qualitative component (Thabane et al., 2010).

The secondary objective was to determine the treatment effect size of the DT treadmill-walking program (DT-TR) compared to a DT recumbent bicycle (DT-RC) program. The working hypothesis was that the group receiving the DT-TR program would demonstrate significantly greater improvement in balance, gait performance, and cognitive measures, compared to the DT-RC program. The following are the reasons for choosing recumbent cycling as the active comparator arm (control group):

1) A previous study has examined effects of dual-task recumbent cycling versus recumbent cycling alone, and demonstrated significantly greater improvement in cognitive performance for the dual-task program as compared to the cycling only exercise (Anderson-Hanley et al., 2012).
2) It provides an aerobic activity comparable to that of treadmill walking, but has minimal stability requirements as compared to walking.
3) In order to provide the participants of both groups with the same types and levels of cognitive games, then participants need to view a computer monitor in order to interact with various cognitive computer games during their respective exercise programs.

## Methods

### Study Design and Setting

This is a mixed methods study using both quantitative and qualitative methods with data from each component collected and analyzed concurrently. The quantitative component was a single blind, pilot, randomized, two-arm, and parallel group-controlled trial comparing participants who received DT-TW with those who received DT-RC. All participants were invited to participate in a semi-structured interview to explore the lived experiences of the study participants. The qualitative findings of participant’s experiences will help to identify; 1) perceived exercise benefits, 2) difficulties with the exercises and using the technologies, and 3) engagement and motivational value of the computer games.

Data collection took place at a community-based exercise center (Reh-Fit center, Winnipeg, MB, Canada). Potential participants based on the eligibility criteria were approached by a member of clinical team by phone and invited to take part in the study. Those interested in participating underwent a screening assessment. Successfully screened participants were randomized by having them choose an envelope at random sequence, each containing a group assignment. All participants signed a consent form.

### Eligibility Criteria

Participant were eligible if they were healthy older adults, aged between 70 and 85 years. Inclusion criteria included: 1) ability to walk at least 400 m without a walking aid, 2) adequate hearing and vision to perform the computer game activities. Exclusion criteria: 1) scored below 25 on the Mini Mental State examination (MMSE) (Grace et al., 1995), 2) history of any neurological disease, 3) uncontrolled hypertension, and 4) other cardiac disease-limiting participant to walk on a treadmill or cycling.

### Procedure

**FIGURE 1:**
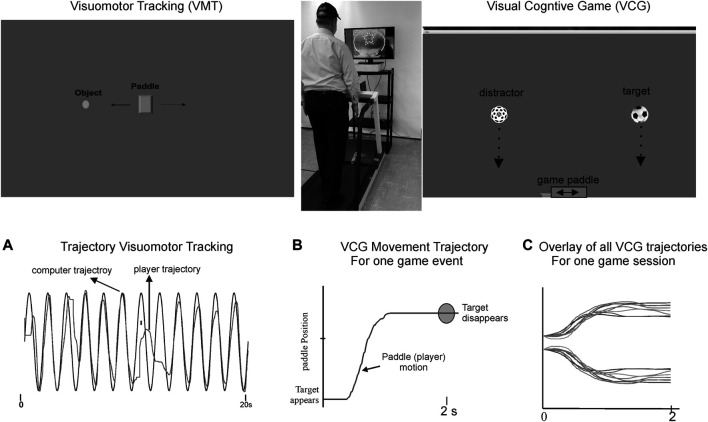
Illustrates the dual-task gaming set-up and snapshots of the visuomotor tracking (VMT) and visuospatial cognitive games (VCG). Top middle picture shows a participant walking on treadmill and playing a computer game. Goal of the VMT game is to track and overlap the game paddle (rectangle object with a moving circle object (computer controlled). Goal of the VCG task is to move the game paddle, to catch target objects (soccer ball), while avoiding distractor objects (dotted sphere). Motion of the rectangle and game paddle are “slaved” to the inertial motion mouse i.e., head pointing movements are used to interact with VM and VCG game activities. Left bottom plot Panel A shows typical movement trajectory of a participant playing the VMT task. It is played for 45 s to obtain several tracking cycles for analysis (e.g., total residual movement error). Panel B presents a game movement trajectory (game paddle coordinates) of one VCG event. Each game event takes 2 s. The VCG game is played for 60 s, and thus 30 game movement responses are recorded. Half of the game events occur in each direction (rightward and leftward movements). All segmented game movement trajectories of one game session (30 game events) are sorted and grouped by direction. Panel C presents the overlay plots of the sorted game movement responses of one participant.

In continuation with previous publications (Szturm et al., 2017), a computer application with the following two assessment modules was used for the DT test conditions: 1) Visuomotor (VM) task, and 2) visuospatial cognitive games (VCG) task. Figure 1 illustrates the gaming set-up for the VM and VCG tasks. An inertial-based (IB) mouse (Gyrations, SMK-Link, United States) was used to interact with the visuospatial cognitive games. To allow for hands-free interaction with game/assessment software, the IB mouse was secured to a participant’s head using a plastic headband. With this simple method, head rotation was used as the pointing device to control the position and motion of the computer game paddle. Therefore, a hands-free computer/game controller was used to interact with the game activities of the assessment software.

### Study Measures

Standing Balance Assessment: The following tasks were performed for 45 ss while standing on a sponge pad; 1) eyes open (EO), 2) performing the VM tracking task, 3) performing the VCG task. As described in previous publications (Desai et al., 2010; Szturm et al., 2015) a force sensor array (FSA) pressure-sensing mat (Vista Medical Ltd., Canada) was used to record vertical foot pressures and to compute center of foot pressure (COP) migration.

Gait Assessment: The following tasks were performed while walking on a treadmill at 0.9 m/s for 1 minute each; 1) walk alone, 2) walking while performing the VM tracking task and 3) walking while performing the VCG task. As described in previous publications (Szturm et al., 2017) treadmill instrumented with a pressure mat (Vista Medical, CA) was used to record vertical foot contact forces and compute spatial-temporal gait variables. Participants walked for 5 min to acclimate to the treadmill prior to testing. The treadmill was equipped with safety side rails in easy reach, and participants were fitted with a safety harness secured above to a support system.

Cognitive ECF was assessed using two validated and standardized neuropsychological tests: Trail Making Test (TMT A and B) (Arbuthnott and Frank, 2000) and Verbal Fluency Test (Woods et al., 2016).

### Intervention

Each participant received a 45-min training program of combined exercise and cognitive activities twice a week for 10 weeks. A physiotherapist trained in using the game controller and computer games delivered the DT-TR and the DT-RC interventions.

DT-TW for experimental group: Each therapy session involved; 1) 5-min warm-up of DT balance exercises with participants standing on a sponge or balance disk while playing various computer games (i.e., various cognitive activities), and 2) treadmill walking while playing various computer games in intervals of 3–5 min and with 1-min rest periods for a total duration of 35 min. The last 5 minutes were used as a cool down period of treadmill walking alone. The initial sponge thickness/density (balance cost), and the treadmill speed were selected so that the participant could perform the DT balance/walking exercises without holding onto the treadmill handrails or the need for any other support (i.e., overhead safety harness).

DT-RC for control group: The program began with a five -minute warm up at a cycling speed at a target heart rate of 40% of maximum, while playing cognitive computer games. A 35 min interval-training program of increasing resistance while playing cognitive computer games followed this. A cycling rate of 50–60 per minute was used. Resistance was adjusted to achieve a target heart rate of 40% of maximum, and gradually increased to 60%. The interval duration was set to 2–3 min with a 1-min rest period. The last 5 minutes was used as a cool down period of cycling alone.

Both groups performed the same visuospatial cognitive activities delivered through interactive computer games. Eight computer games were selected for each participant from a pool of 20 games purchased from Big Fish Games (www.bigfishgames.com). The computer games involved goal-directed cognitive activities including 1) visual search and tracking of multiple targets, 2) speed accuracy requirements, 3) presence of distracters, 4) matching tasks, and 5) working memory. Supplementary Appendix S1 for a list of the games and brief description of the executive cognitive tasks.

### Qualitative Interviews

On completion of the exercise program, all participants were invited to take part in a 30-min semi-structured interview. The following open-ended questions were asked of each participant:

1. When you agreed to participate, how did you hope you would benefit from the therapy program?
2. What did you like and dislike about the therapy program?
3. What did you think about the exercises and the computer games you were asked to play?
4. Did you feel that this therapy program helped you?
5. If you were provided with the right settings, would you continue with these exercises?

A research assistant who was blinded to the intervention received by the participant conducted the interviews. A second person was present to record in writing all responses. Participants were encouraged to describe and explain their ideas, thoughts, and opinions. The analytical framework of interpretive description was used for thematic interpretation (Teodoro et al., 2018). The written response transcripts were initially read by one researcher who developed the coding system by paraphrasing, generalizing, and abstracting the written responses of each interview. A second researcher scrutinized the coded data, and identified any additional unique responses. The two researchers then met to compare their analyses and to resolve disagreements and a final coded response system was produced organized into final themes.

### Data Analyses

Qualitative analysis: Written responses of the participants were reviewed and analyzed by two physiotherapists using the interpretive descriptive method (Thorne et al., 1997). Participants’ interview responses were coded, and similar codes were grouped into unique themes. These themes were used to describe the data, and to illustrate a range of ideas, experiences, and viewpoints.

Quantitative analysis: As per the methods of (Szturm et al., 2015) standing balance performance was quantified by computing the root mean squared (RMS) COP excursions in the anterior-posterior (AP) and medial-lateral (ML) directions for each task (i.e., eyes open, VCG and VM).

The following spatiotemporal gait variables were determined from the treadmill COP recordings; 1) average Step length and average step time, and 2) coefficient of Variation (COV) of step length, and COV step time. These were computed from; 1) 45 consecutive steps of the walk only (WO) trials, 2) 45 consecutive steps of the VCG dual-task walking trials, and 3) 30 consecutive steps of the VM dual-task walking trials. The gait variables have shown good to excellent test-retest reliability (Szturm et al., 2017). Average and COV of step time and step length were determined for right and left gait variables. Statistical analysis (*t*-test) demonstrated no significant difference in means of right and left gait variables, and therefore only the right-side gait variables were reported in the results.

Normality of the data was assessed using the Shapiro-Wilks test. This test revealed a normal distribution *p* < 0.01 for all outcome measures. A two-way repeated measures ANOVA was used to examine the effects of time (pre–and post-intervention) group (DT-TR and DT-RC) and interaction of time*group of the balance, gait, and cognitive outcome measures. Post hoc pairwise comparison with Bonferroni corrections were then conducted. Effect size was calculated using Cohen *d* (Kuspinar et al., 2012), taking the difference in the mean change in the primary outcome between the intervention and control groups and dividing it by the initial pooled standard deviation. Data were analyzed using SPSS (Version 22) (SPSS Science, Chicago).

## Results

Twenty-six participants were recruited and screened for the study in a time of 8 months. There were two-dropouts after the initial screening assessment, before randomization. They both stated that the program was not to their liking. Two participants in the DT-TR group dropped out after two training sessions - one experienced considerable hip and knee pain and could not continue the training; the other participant did not feel comfortable with the DT-TR program. Eleven participants in the DT-TW program and 11 in the DT-RC completed the 10-weeks exercise programs and attended all 20 sessions.

Participant characteristics at baseline are presented in Table 1. There were no significant differences between groups in any baseline measure.

**TABLE 1:** Group demographic and clinical data.

Variables	DT-TR (*n* = 11)	DT-RC (*n* = 11)
Age (years)	75.5 ± 3.1	76.1 ± 3.9
Gender ratio (Males: Females)	6:5	5:6
Mini Mental Status Examination	28.7 ± 1.0	29 ± 0.44
Gait speed (m/s)	1.13 ± 0.1	1.03 ± 0.6
Six-minute walking test (215.16 m./lap)	584.2 ± 48.9	642.55 ± 26.5
Five times sit to stand test (second)	10.18 ± 2.5	10 ± 0.8

DT-TR: dual task treadmill training; DT-RC: dual task recumbent cycling.

### Qualitative Findings

Seven out of eleven participants in each group agreed to be interviewed. The following four themes emerged from the qualitative analysis:

1. Reasons for participation: Most participants in both groups commented that fall was a major concern and participated in the hope that the DT exercise programs would improve their balance and confidence in walking outdoors. Four participated because staff at the exercise center recommended the program. Three of the participants in the DT-RC group commented that they would have preferred to do the DT-TR program as they felt it would train gait better than cycling.
2. Difficulties with using the technologies: All participants commented that they have not done this type of exercise before. Ten of the participants (6 in DT-TR and four in DT-RC) said that they have not played computer games before. Most participants in both groups commented that it was difficult at first to play the games while exercising, and that the games required lots of concentration. All participants commented that it was good the therapist was there to help them with playing the games. Most of the participants in both groups commented that the 45 min exercise duration was plenty and that they were tired after completing the exercise sessions. Three of the participants in the DT-RC group and five participants in the DT-TR group commented it was difficult at first to use the IB mouse, and said that it was annoying with the game paddle/cursor drifted to one side of the computer monitor. At times, the mouse would drift away to one side of the screen because of sudden movements of the head. This was frustrating because once drifting occurred, they could not control the game paddle in one direction, and they did not like losing in the games. In the first few exercises session they needed help from the physiotherapist before they became comfortable and proficient in using head rotation to control the game paddle and to learn how to minimize the drifting. This was more a problem for those walking on the treadmill as compared to participants who were seated. Two participants in both groups commented that they did not have any problems with the games; most of the participants were amazed to find out how they could manage to perform the exercises and the games simultaneously.
3. Engagement and motivational value of the computer games. Most participants in both groups commented that the games kept them engaged, and that they liked the variety; each game was different and had different challenges and difficulty levels. All participants became aware of the various mental features of the games. Most participants found some games interesting and fun, but some games were disliked. The preferences varied among the participants. Three participants (1 in DT-RC and 2 in DT-TR) did not like any of the games. Most participants commented that focusing on playing the games made the time pass quicker.
4. Other Comments: Many of the participants expressed that they would keep doing it, provided it was available at the exercise facility. Most participants in both groups commented that they would want their program to be monitored as they felt they would not be able to progress the exercises alone. Six of the participants (4 in the DT-TR and 2 in the DT-RC group) commented that they would recommend the program to others at the exercise center, but also commented that a therapist would be required to help start the program and select the cognitive games.

### Quantitative Findings

**FIGURE 2:**
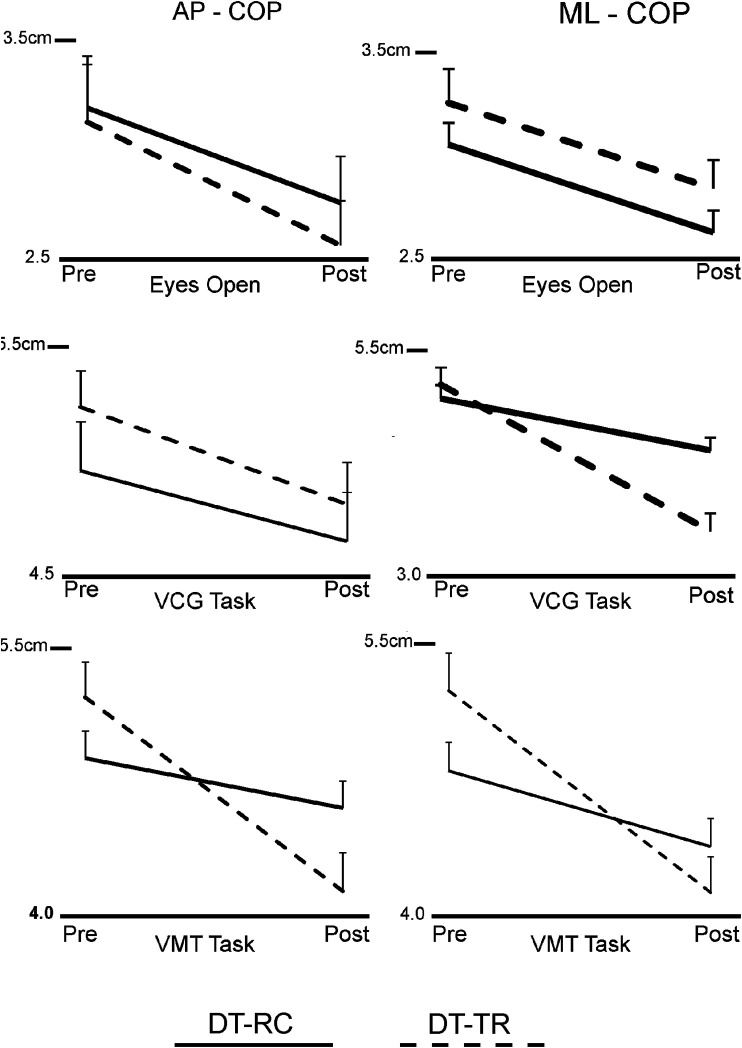
Line plots of group means and standard error of means (SEM) of COP excursion (RMS), in the anterior-posterior direction (AP-COP) and in the Medio-lateral direction) ML-COP), Pre- and Post-intervention.

Group means and standard error of means (SEM) of COP excursion (RMS) are presented in Figure 2. Table 2 presents ANOVA results for AP and ML COP excursion. There was a significant decrease in COP excursion in both AP and ML directions from pre-to post-intervention. This was the case for EO and DT test conditions. There was no Group effect, but there was a significant time*group interaction for the DT balance conditions in the ML direction, and for VCG in the AP direction. As seen in Figure 2, the magnitude of change of COP excursion pre-to post-intervention were significantly greater (Time*Group interaction) for the DT-TR group compared to the DT-RC group. This was the case for VM and VCG conditions as well.

**TABLE 2:** Results of ANOVA for the root mean squared (RMS) center of pressure (COP) excursions in the anterior-posterior and medial-lateral directions.

Outcome	Time F-statistics, p-value, d	Group	Time*Group
		F-statistics, p-value, d	F-statistics, p-value, d
**AP-RMS**			
Eyes Open	23.1, 0.0001, 0.56	0.05, 0.83, 0.003	0.39, 0.53, 0.021
VCG	43.4, 0.0001, 0.7	0.33, 0.6, 0.018	1.11, 0.3, 0.058
VMT	125.85, 0.0001, 0.87	0.026, 0.8, 0.001	43.94, 0.0001, 0.70
**ML-RMS**			
Eyes Open	187.4, 0.0001, 0.917	2.73, 0.11, 0.13	0.012, 0.91, 0.001
VCG	353.64, 0.001, 0.95	0.086, 0.77, 0.055	109.73, 0.0001, 0.85
VMT	525.5, 0.0001, 0.96	2, 0.18, 0.1	69.75, 0.0001, 0.795

VCG: visuospatial cognitive games; VMT: visuomotor tracking, d = Cohen d (effect Size).

**FIGURE 3:**
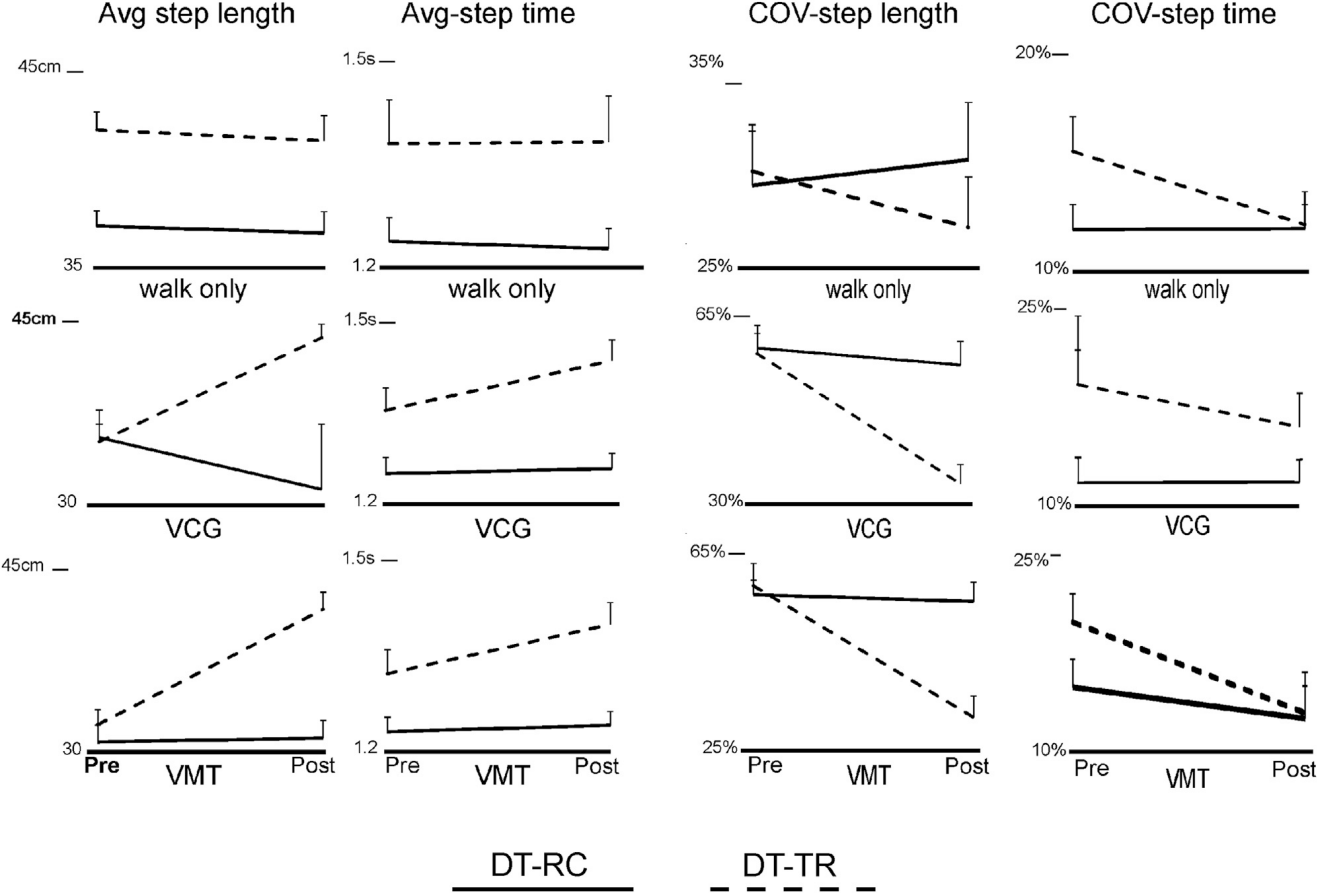
Line plots of group means and SEM of Step length and step time, both average values (Avg) and coefficient of variation (COV), Pre- and Post-intervention *Y*-axis scale for step length is centimeters (cm) and for step time is seconds (s).

Group means and SEM of the spatio-temporal gait variables are presented in Figure 3. Table 3 presents ANOVA results for the spatiotemporal gait variables (average and COV). The results for walk alone showed that there was no Time or Time*Group effects in gait performance (average or COV). There was a significant Time and Time*Group Effect on average step length and step time for the dual task walking conditions, VM and VCG. There was a significant Time and Time*Group effect on COV-SL and COV-ST during the dual-task VCG walking condition, and on COV-SL during the dual-task VM walking condition. There was also a significant Time*Group effect on COV-ST during the VM walking condition. As seen in Figure 3 (average values) and four (COV values) the magnitude of change in step length and step time pre-to post-intervention were significantly greater (Time*Group interaction) for the DT-TR group compared to the DT-RC group. This was the case for VM and VCG conditions; significant increase in average step length and a significant decrease in COV. Post hoc analysis revealed that there were no significant changes in average or COV step length/time pre to post intervention for the DT-RC group.

**TABLE 3:** Results of ANOVA for average (Avg.) and coefficient of variation (COV) gait variables, Step Length and Step Time.

Outcome	Time F-statistics, p-value, d	Group F-statistics, p-value, d	Time*Group F-statistics, p-value, d
**Walk Only**			
Avg. Step Length	0.63, 0.43, 0.03	10.8, 0.004, 0.35	0.03, 0.86, 0.002
Avg. Step Time	0.05, 0.81, 0.003	3.2, 0.08, 0.14	0.14, 0.70, 0.007
**VCG**			
Avg. Step Length	31.8, 0.0001, 0.61	10.34, 0.004, 0.34	30.4, 0.0001, 0.60
Avg. Step Time	28.7, 0.0001, 0.58	9.3, 0.006, 0.32	19, 0.0001, 0.48
**VMT**			
Avg. Step Length	33.99, 0.0001, 0.63	13.1, 0.002, 0.4	29.48, 0.0001, 0.59
Avg. Step Time	21.3, 0.0001, 0.51	8.1, 0.01, 0.3	12.68, 0.002, 0.38
**Walk Only**			
COV Step Length	0.04, 0.34, 0.04	0.66, 0.42, 0.03	6.9, 0.01, 0.25
COV Step Time	0.16, 0.69, 0.008	1.8, 0.2, 0.08	0.24, 0.62, 0.012
**VCG**			
COV Step Length	62.5, 0.0001, 0.75	0.29, 0.59, 0.02	36.71, 0.0001, 0.64
COV Step Time	4.7, 0.04, 0.19	1.7, 0.2, 0.08	4.91, 0.03, 0.19
**VMT**			
COV Step Length	175, 0.001, 0.89	1.1, 0.24, 0.06	142, 0.001, 0.88
COV Step Time	5.56, 0.02, 0.21	0.02, 0.88, 0.001	2.6, 0.12, 0.115

VCG: visuospatial cognitive games; VMT: visuomotor tracking; d = Cohen d (effect Size).

**FIGURE 4:**
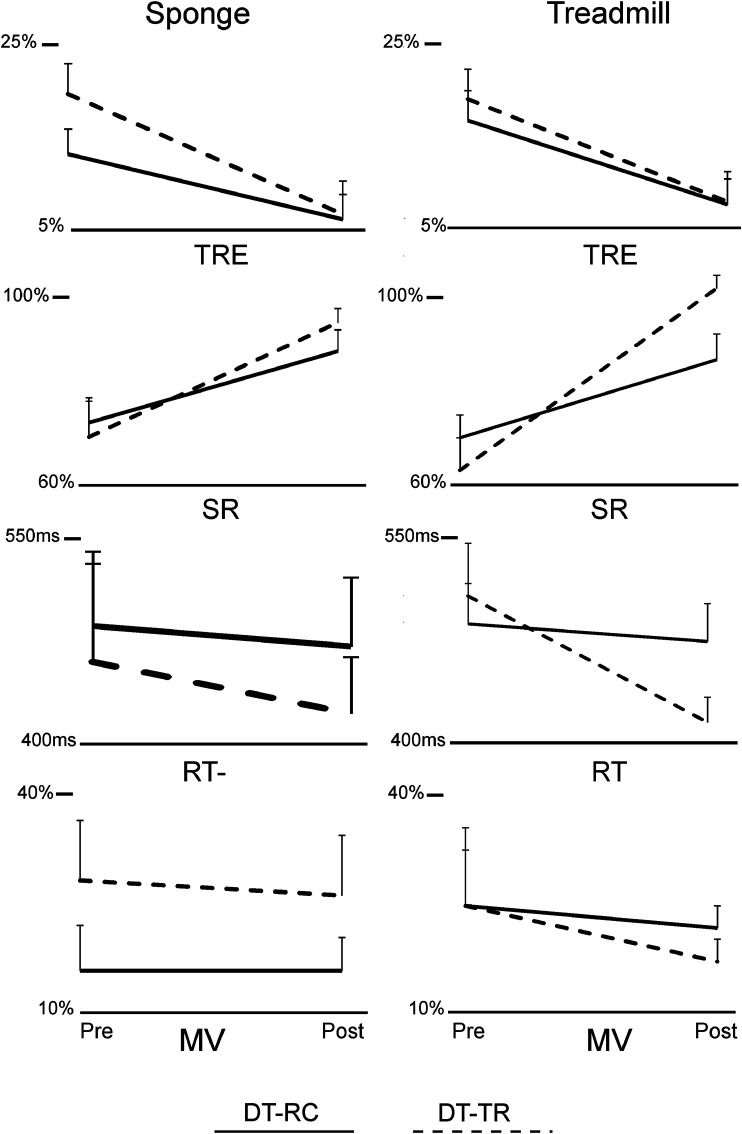
Line plots of group means and standard error of means (SEM) of visuomotor tracking (VMT) and visuospatial cognitive games (VCG) performance measures, Pre- and Post-intervention. Total Residual Error (TRE), Success Rate (SR), Response Time (RT) and Movement Variation (MV). *Y*-axis scale for TRE and MV is %display height/width, respectively, for SR is the % of total targets caught, and for Response time is seconds (s).

**FIGURE 5:**
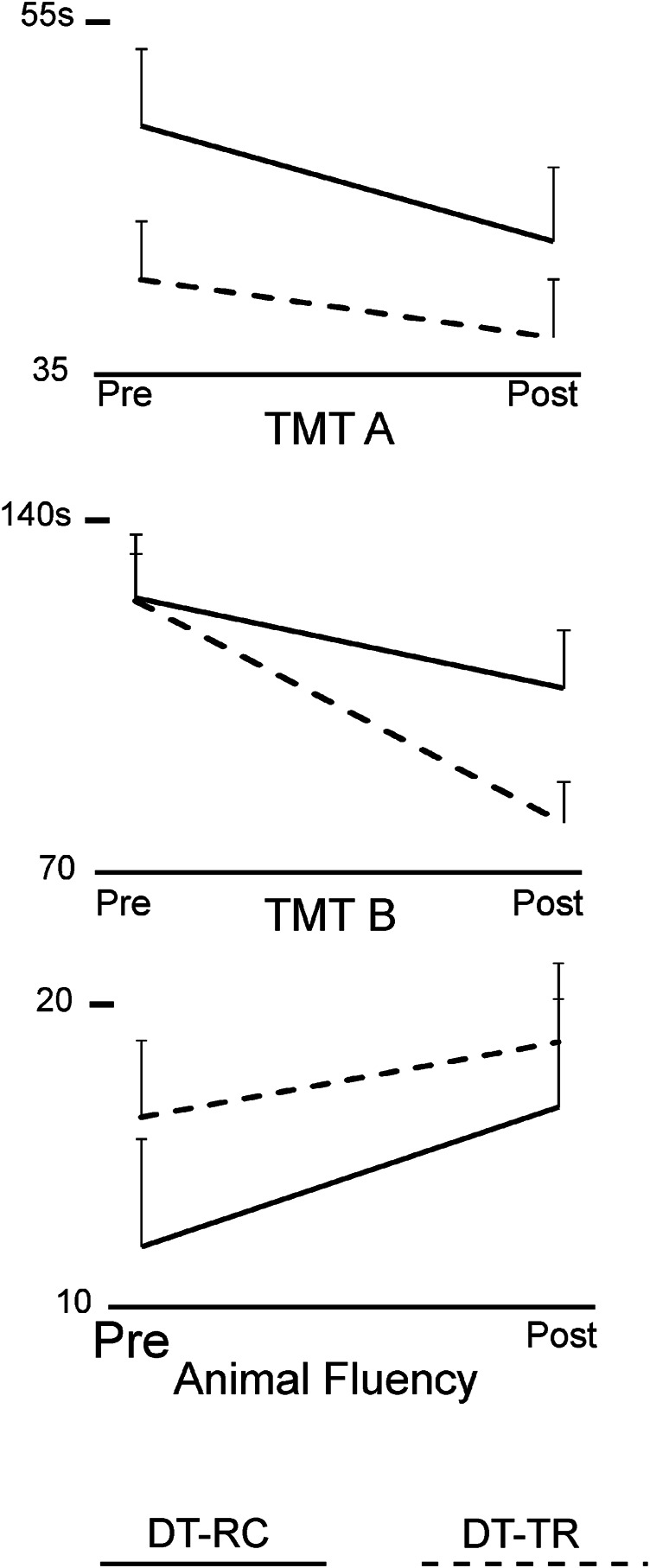
Line plots of group means and SEM of TM-A, TMT-B and Verbal Fluency Test scores, Pre- and Post-intervention.

Group means and SEM of VM and VCG performance measures are presented in Figure 4. Table 4 presents ANOVA results for the VM and VCG performance measures. As presented in Table 4 and evident from Figure 5, there were significant increases in VM performance pre to post intervention in both the DT-TR and DT-RC groups. This was the case when standing on sponge surface and during treadmill walking. There was a significant improvement in Success Rate and Movement Variation, and a significant decrease in Response Time from pre to post intervention in both the DT-TR and DT-RC groups. This was the case when standing on a sponge surface and during treadmill walking. There was no Group or Time*Group effect on Success rate or Response Time, but there was a significant Time*Group interaction for movement variation. A greater change pre-to post-intervention for the DT-TR group compared to the DT-RC group.

**TABLE 4:** Results of ANOVA for Visuomotor tracking performance measure, Total Residual Error and for Visual Cognitive Game performance measures, Success Rate, Response Time and Movement Variation.

Conditions	Time F-statistics, p-value, d	Group F-statistics, p-value, d	Time*Group F-statistics, p-value, d
**Total Residual Error**			
Sponge	10.656, 0.004*, 0.35	1.199, 0.28, 0.06	1.243, 0.27, 0.06
Treadmill	21.975, 0.00*, 0.52	1.597, 0.22, 0.07	0.324, 0.57, 0.02
**Success Rate**			
Sponge	6.49, 0.02*, 0.25	0.01, 0.92, 0.001	0.001, 0.97, 0.001
Treadmill	32.90, 0.00*, 0.62	0.78, 0.38, 0.04	1.619, 0.21, 0.07
**Response Time**			
Sponge	6.03, 0.02*, 0.23	2.77, 0.11, 0.12	0.039, 0.84, 0.00
Treadmill	6.10, 0.02*, 0.23	2.84, 0.10, 0.12	0.23, 0.64, 0.01
**Movement Variation y**			
Sponge	3.43, 0.07*, 0.15	2.79, 0.11, 0.12	4.45, 0.05*, 0.18
Treadmill	6.68, 0.01*, 0.25	1.26, 0.27, 0.06	6.00, 0.02*, 0.23

d = Cohen d (effect Size).

Group means and SEM of the neuropsychological test scores are presented in Figure 5. Table 5 presents ANOVA results for the neuropsychological test scores. There was a significant improvement in TMT-A and TMT-B performance pre to post intervention in both groups, and Time*Group interaction was not significant. There was no significant Time or Time*Group effects for the Verbal Fluency Test scores.

**TABLE 5:** Results of ANOVA for trail making test (TMT) and verbal fluency test.

Measures	Time F-statistics, p-value, d	Group F-statistics, p-value, d	Time*Group F-statistics, p-value, d
TMT-A	8.32, 0.01*, 0.29	5.29, 0.03*, 0.21	2.96, 0.10, 0.13
TMT-B	10.27, 0.00*, 0.34	0.90, 0.35, 0.04	1.83, 0.19, 0.08
Verbal Fluency Test	5.89, 0.02*, 0.23	0.60, 0.45, 0.03	0.54, 0.47, 0.03

d = Cohen d (effect Size).

## Discussion

The purpose of the present study was to evaluate the feasibility of conducting an RCT using the DT treadmill and recumbent cycle programs and to obtain preliminary data of the effect size of the program in older adults. The general findings from the interviews were that participants found that dual-task program was well conducted and that the difficulty level of both exercise programs was challenging. Twenty-two of the 24 participants who entered the program completed all 20 exercise sessions. There are several likely reasons for the high compliance rates: 1) Continued guidance, instructions and assistance were provided to the participants to ensure that they understood the games and were provided games with appropriate difficulty levels, 2) There was ample time during each session to address any questions about the basis of the dual-task approach, the selection of cognitive activities in the computer games and the training intensity, 3) As reported by most of the participants, the interactive commercial computer games used in the present study were engaging and fun, and 4) The program was accessible and there was no added cost.

A variety of 20 commercial computer games were used. Selection of these computer games was based on the information processing demands imposed by the game. Various game genres were used which required visual search, visual tracking, trajectory estimation, distractors, time constraints, matching by color/shape, working memory, and precision pointing movements. Matching and shooting games required participants to use a small wireless hand-held optical mouse with a left mouse button to press when needed. The variety of available games helped participants to remain engaged during their exercise program. Apart from the variety in the cognitive challenge, increasing cycle resistance and treadmill speed was used to progress the DT walking exercise.

Balance performance improved post-intervention in both groups under challenging compliant surface conditions while performing visuomotor and cognitive dual tasks. Other studies have also reported improvement in various measures of standing balance following a cycling exercise program (Thorne et al., 1997) however the improvement in dual-task balance performance observed in the present study were significantly greater in the DT-TR group compared to the DT-RC group. Although there was a significant training effect on balance performance observed for the DT-RC group, there was no improvement in gait performance (either average or COV measures). Previous studies have shown that various exercise programs that results in improvement in standing balance capabilities do not necessarily transition into improvement in walking capabilities (Mangione et al., 1999; Szturm et al., 2011; Conradsson et al., 2015). For example, (Szturm et al., 2011; Szturm et al., 2011) has examined the effect of interactive computer game exercises performed in standing by older adults, and results showed a significant improvement in standing balance performance when tested on a compliant sponge with eyes open and closed. However, there was no significant improvement in gait speed or spatial-temporal gait variables.

As expected, there was a significant improvement (pre-to post-intervention) in gait performance measures (average and COV) for the DT-TR group, and medium to large effect sizes were observed. There was no significant improvement in gait performance pre-to post-intervention in the DT-RC group. For example, gait variability decreased on average by 30–40% in the DT-TR group, whereas gait variability decreased by less than 10% in the DT-RC group. Gait variability measures are important outcome measures as they reflect gait stability (Conradsson et al., 2015) and are independent predictors of falls (Ahmadi et al., 2019). Other DT exercise programs (Callisaya et al., 2011; Gregory et al., 2017; Conradsson and Halvarsson, 2019). have reported improved standing balance and gait performance of older adults. The cognitive tasks used in these studies (Dorfman et al., 2014; Gregory et al., 2017; Conradsson and Halvarsson, 2019). included walking while talking, verbal fluency, serial subtraction, or phoneme monitoring. The current study extends these results to include visuomotor and visuospatial cognitive activities. Besides language, arithmetic, and recall tasks, processing the spatial relations and features between objects, tracking moving objects and visual attention are important ECF to consider in the analysis of dual-task interference on mobility (Nagamatsu et al., 2009). Following the principle of neural overlap (Crockett et al., 2017), dual-task interference should be greatest when the cognitive and motor tasks engage the same neural circuits and processing resources, e.g., Visual-spatial processing. The VMT task used in the present study requires real-time on-line visual feedback of the relative positions of two objects. The VMG task requires visual search to locate target objects and cognitive inhibition to avoid distractors. The VM and VCG tasks required precision head-pointing movement. Many real-life tasks involve head movements to track and locate various object and for information processing of what is being seen. The increased visuospatial processing necessary to maintain walking rhythm and to correct for any drifting on the treadmill would compete for resources required to perform the VMT and VMG tasks, and vice versa.

The present results show a significant intervention effect in visuomotor and cognitive game performance with medium to large effect sizes when assessed during treadmill walking. Improvement was similar in both the DT-W and DT-RC groups, i.e., Time*Group interaction was not significant. A significant improvement in both groups with medium effect sizes was also observed for TMT-A and TMT-B scores. Other studies also report positive small-to-medium effects of combined cognitive and physical exercise interventions on ECF in older adults (Nagamatsu et al., 2009; Anderson-Hanley et al., 2012; Gregory et al., 2017). One common outcome measure in these studies is the Trail Making test. In the study of (Anderson-Hanley et al., 2012; Anderson-Hanley et al., 2012) they report that individuals who performed a cyber-cycle exercise program achieved greater improvement in performance on the Trail Making Test (color version) as compared to a group who performed a cycling only exercise program.

Treadmill-based interventions involve repetitive stepping and dynamic stability requirements that are comparable to the demands of usual gait. Treadmills can be easily equipped with a video monitor, and therefore one has easy access to digital media. Many common and modern computer games have a broad range of executive cognitive content. Treadmill training also prevents the ability to reduce gait speed as a strategy to prioritize gait stability when faced with divided attention. Also important are the safety features available when using treadmills, i.e., front and side handrails, kill-switch to stop the treadmill, and body weight support systems which prevent falling or excessive drifting.

Moreover, to measures of structure and function (e.g., Cop excursion, spatiotemporal gait variables and cognitive game outcome measures) future powered randomized control trials should also include outcome measures such as fall incidence (Lipardo and Tsang, 2020) over a 1-year time period, level of physical activity (Fuller et al., 2020), and life-role participation (Gardner, 2014) to validate the findings. In order to better understand the effectiveness of the gaming platform to increase the intrinsic and extrinsic level of motivation and engagement, patients’ theories of motivation (such as achievement goal theory) (Palmer et al., 2017) can also be used in future studies.

### Limitations

One limitation relates to how individuals spontaneously prioritize their attention between the walking and the tracking/cognitive tasks during the DT testing. It is possible that walking was prioritized, and participants did not attend to or process the computer tracking/cognitive information on the computer monitor. Alternatively, the information was received and processed but the performance was affected due to dual-task interference. Modest performance levels were observed for both tracking and cognitive game tasks during walking so the participants were attending to and processing the information, they saw on the display. But they may have stopped intermittently for a few seconds and prioritize locomotor processing. Participants played different games, which had different cognitive loads. This may have introduced a confounding variable. It is not known whether one group played more difficult games than the other did. The types of games used for DT training would need to be controlled in future studies Another limitation is that the sample only included people who were attending exercise classes on a regular basis, and only from one exercise facility.

## Conclusion

The computerized dual-task protocol presented in this study broadens the type of standardized visuospatial cognitive activities for use with treadmill training that has previously been reported. The game-assisted treadmill training intervention was found to be highly feasible. Although some difficulties with the technology were reported, the findings demonstrate feasible trial procedures and acceptable DT task-oriented training with a high compliance rate and positive outcomes. These findings and the theoretical evidence direct the next phase of a full-scale randomized controlled trial (RCT).

## Data Availability

The original contributions presented in the study are included in the article/Supplementary Material, further inquiries can be directed to the corresponding author.
