# Supplementary material for: Dual-Task Training Program for Older Adults: Blending Gait, Visuomotor and Cognitive Training
Source: Front Netw Physiol. 2021 Sep 29;1:736232. doi: 10.3389/fnetp.2021.736232 (PMC10013153; doi:10.3389/fnetp.2021.736232)
Supplement: Supplementary file 1 [file DataSheet1.docx]

**Appendix 1.** Computer game details. (See www.Bigfishgames. Com) Note matching and shooting games require participants to use a small wireless hand-held clicker with a left mouse button to press when needed.

| **Big Fish Game** | **Axis Play** | **Start Difficulty** | **Type** | **Clicker** | **Precision** | **Background** | **Distractor** | **Executive Function** |
| --- | --- | --- | --- | --- | --- | --- | --- | --- |
| Abundante | Horizontal | Difficult | Match 3 | Yes | Moderate | Low Optokinetic | No | Matching and Puzzle Solving |
| action ball | Horizontal | Moderate | Brick Buster | No | Moderate | High Optokinetic | Yes | Visual Tracking & Spatial |
| Acqua Ball | Horizontal | Easy | Brick Buster | No | Low | Medium optokinetic | Yes | Visual Tracking & Spatial |
| Astrobugs Revenge | Horizontal | Difficult | Match 3 | Yes | High | Medium optokinetic | No | Matching |
| Ark Light | Variable | Moderate | Shooting | Yes | Moderate | Medium optokinetic | Yes | Search and Select |
| Birds Town | Horizontal | Moderate | Match 3 | Yes | High | Low Optokinetic | No | Matching |
| Brave Piglet | Vertical | Moderate | Shooting | Yes | High | Low Optokinetic | Yes | Visual Tracking & Spatial |
| Bricks of Egypt | Horizontal | Easy | Brick Buster | No | Variable | Low Optokinetic | Yes | Visual Tracking & Spatial |
| Butterfly Escape | Horizontal | Moderate | Match 3 | Yes | High | Low Optokinetic | Yes | Visual Tracking & Spatial |
| Chicken Invaders | Variable | High | Shooting | Yes | Moderate | High Optokinetic | Yes | Search and Select |
| Digby Donuts | Horizontal | Moderate | Catch &Sort | Yes | Moderate | Low Optokinetic | No | Search precision Sort |
| Feeding Frenzy | Variable | Difficult | Aim and Move | No | High | Low Optokinetic | Yes | Visual Tracking & Spatial |
| Invadazoid | Horizontal | Difficult | Brick Buster | No | High | Moderate Optokinetic | Yes | Visual Tracking & Spatial |
| Jar of Marbles | Horizontal | Easy | Match 3 | Yes | Medium | Low Optokinetic | No | Matching Three , aligning |
| Jet Jumper | Variable | Difficult | Driving Game | Yes | High | High Optokinetic | Yes | Visual Tracking and Driving |
| Luxor 3 | Horizontal | Moderate | Match 3 | Yes | High | Moderate Optokinetic | Yes | Match 3, Aligning |
| Luxor HD | Horizontal | Moderate | Match 3 | Yes | High | Moderate Optokinetic | Yes | Match 3, Aligning |
| Reaxion | Horizontal | Moderate | Brick Buster | No | Variable | Moderate Optokinetic | Yes | Visual Tracking & Spatial |
| Ricochet Recharge | Horizontal | Moderate | Brick Buster | No | High | Moderate Optokinetic | No | Visual Tracking & Spatial |
